# *AcMYB10* Involved in Anthocyanin Regulation of ‘Hongyang’ Kiwifruit Induced via Fruit Bagging and High-Postharvest-Temperature Treatments

**DOI:** 10.3390/genes15010097

**Published:** 2024-01-14

**Authors:** Min Yu, Jinyu Xiong, Kun Dong, Xin Quan, Hao Guo, Junwei Huo, Dong Qin, Yanchang Wang, Xuemei Lu, Chenqiao Zhu

**Affiliations:** 1 College of Life Science, Northeast Agricultural University, Harbin 150030, China; 2 College of Horticulture & Landscape Architecture, Northeast Agricultural University, Harbin 150030, China; 3 Horticulture Branch, Heilongjiang Academy of Agricultural Sciences, Harbin 150040, China; 4 Key Laboratory of Biology and Genetic Improvement of Horticultural Crops (Northeast Region), Ministry of Agriculture and Rural Affairs, Harbin 150030, China; 5 Key Laboratory of Plant Germplasm Enhancement and Specialty Agriculture, Wuhan Botanical Garden, Chinese Academy of Sciences, Wuhan 430074, China

**Keywords:** *Actinidia chinensis*, transcription factor, fruit coloration, MYBs

## Abstract

Light and temperature are key factors influencing the accumulation of anthocyanin in fruit crops. To assess the effects of fruit bagging during development and high post-ripening temperature on ‘Hongyang’ kiwifruit, we compared the pigmentation phenotypes and expression levels of anthocyanin-related genes between bagged and unbagged treatments, and between 25 °C and 37 °C postharvest storage temperatures. Both the bagging and 25 °C treatments showed better pigmentation phenotypes with higher anthocyanin concentrations. The results of the qRT-PCR analysis revealed that the gene expression levels of *LDOX* (leucoanthocyanidin dioxygenase), *F3GT* (UDP-flavonoid 3-O-glycosyltransferase ), *AcMYB10*, and *AcbHLH42* were strongly correlated and upregulated by both the bagging treatment and 25 °C storage. The results of bimolecular fluorescence complementation and luciferase complementation imaging assays indicated an interaction between AcMYB10 and AcbHLH42 in plant cells, whereas the results of a yeast one-hybrid assay further demonstrated that *AcMYB10* activated the promoters of *AcLODX* and *AcF3GT*. These results strongly suggest that enhanced anthocyanin synthesis is caused by the promoted expression of *AcLODX* and *AcF3GT*, regulated by the complex formed by *AcMYB10*–*AcbHLH42*.

## 1. Introduction

Kiwifruit (*Actinidia chinensis*), a woody vine-borne edible berry belonging to the Actinidiaceae family, is one of the most valuable fruit crops because of its diverse nutritional and health benefits for humans [1]. The *Actinidia* genus encompasses approximately 75 species, exhibiting a diverse array of colors in both the skin and flesh, which is primarily determined by the accumulation of chlorophyll, carotenoids, anthocyanins, and their various combinations [2]. The current commercial kiwifruit (*A. chinensis*) cultivars available on the market are commonly categorized into green-, yellow-, and red-flesh varieties [3]. Among them, ‘Hongyang’, the most prominent and renowned, red-fleshed kiwifruit cultivar in China, exhibits a star-shaped accumulation of anthocyanins in the inner pericarp. This unique characteristic increases its appeal to customers and breeders compared with green-fleshed varieties due to the widely accepted biofunctionality of anthocyanins [4,5]. In cultivation practice, various environmental factors influence the accumulation of anthocyanins in fruit, such as temperature, levels of plant nutrients, shading, and concentrations of endogenous hormones [6], which create challenges in the cultivation of red-fleshed kiwifruit.

Fruit bagging is an important pomological technique employed during fruit development to optimize the visual appearance and internal quality of fruits by effectively manipulating the microenvironment surrounding the fruit, including controlling light intensity, temperature, and humidity levels. Fruit bagging also serves as a protective measure against abiotic and biotic stressors [7,8]. Given the extensive research on the photoregulation of anthocyanin production in the plant kingdom [9], fruit bagging has emerged as an effective technique for enhancing the peel pigmentation in various fruits, including apples [10,11], peaches [12], grapes [13], and pears [14]. Moreover, fruit bagging management is common in the production of kiwifruit, aiming to increase fruit quality. It has been applied with green-fleshed ‘Jinkui’ (*A. chinensis*) [15], yellow-fleshed ‘Jinyan’ (*A. chinensis*) [16], as well as *A. eriantha* lines “G19”, “G21”, and “G28” [17]. In ‘Hongbaoshixing’ (a whole-fruit pigmented *A. arguta* cultivar), bagging treatment throughout fruit development substantially restricted the proper coloration of peel [18]. Although fruit bagging can enhance the fruit storability, flesh color, as well as flavonoid and anthocyanin contents of ‘Hongyang’, the underlying mechanism of these effects remains poorly understood [19,20,21]. Low temperatures stimulate anthocyanin accumulation in the plant kingdom [22,23], whereas high temperatures suppress anthocyanin accumulation [24,25]. In terms of the cultivation practices used for ‘Hongyang’, the fruits harvested in warmer temperature regions commonly exhibit poor flesh pigmentation compared with those harvested in cooler areas [26]. Although our previous study revealed that 25 °C might serve as the threshold for anthocyanin accumulation during the fruit-ripening process of ‘Hongyang’ on the tree, and we hypothesized that the kiwifruit-ripening process could tolerate temperatures as high as 35–40 °C, the impact of relatively high temperatures on postharvest anthocyanin accumulation remains unclear, as most commercial kiwifruit cultivars require early harvesting, with subsequent postharvest ripening.

In terms of anthocyanin biosynthesis, the key structural genes have been extensively documented, most of which have been profiled, mined, isolated, cloned, and function-verified by various multi-omic approaches and molecular methods. These genes include *CHS* (encoding chalcone synthase), *F3H* (favanone 3-hydroxylase), *DFR* (dihydrofavonol 4-reductase), *LDOX/ANS* (leucoanthocyanidin dioxygenase), and *UFGT* (UDP-flavonoid 3-O-glycosyltransferase) [27,28,29,30,31]. Since the early 2000s, accumulating evidence has indicated that changes in the expression of these structural genes, which are regulated by transcription factors (TFs), are responsible for light- and temperature-induced anthocyanin accumulation in fruit crops [24,32]. Among the key TFs involved in regulating anthocyanin biosynthesis, R2R3-MYB genes are the most crucial family regulating fruit coloration through functionating with the MYB–bHLH–WD40 complex [33,34]. In kiwifruit, more than 150 MYBs have been documented [35,36]. Among them, *AcMYB110* [37], *AcMYBF110* [2], *AcMYB75* [23], *AcMYB10* [23,36], and *AcMYB123* [38] are involved in the regulation of anthocyanin synthesis. Moreover, in our previous study, we demonstrated that *AcMRP* and *AcMYB1* mediate the downregulation of anthocyanin biosynthesis in ‘Hongyang’ under high-temperature conditions [26]. Additionally, as a related paralog of *AcMYB1*, we verified that *AcMYB10* is a key regulator participating in light- and temperature-induced anthocyanin biosynthesis in vitro [36]. Therefore, whether *AcMYB10* is involved in the regulation of anthocyanin synthesis induced by bagging and high temperatures during the post-ripening period is worth investigating. This investigation contributes towards achieving a comprehensive understanding of anthocyanin accumulation in ‘Hongyang’ kiwifruit, thereby providing a theoretical foundation for molecular breeding, standardized cultivation practices, and postharvest technologies aimed at enhancing flesh coloration.

To evaluate the impact of bagging treatment and high postharvest temperatures on anthocyanin synthesis in ‘Hongyang’, we conducted bagging treatments during fruit development and subjected the fruits to 37/25 °C treatments during the post-ripening period. Subsequently, we compared the pigmentation of the fruits and expression levels of the relevant genes. Additionally, we performed bimolecular fluorescence complementation (BiFC) and bimolecular luminescence complementation (BiLC) assays to investigate the interaction between AcMYB10 and AcbHLH42. Furthermore, a yeast one-hybrid (Y1H) assay was employed to confirm the promoter achievability of *AcMYB10* and *AcbHLH42* on *AcLODX* and *AcF3GT*. This study provides valuable insights into elucidating the mechanism underlying light- and temperature-regulated anthocyanin biosynthesis, as well as developing pre- and postharvest treatment strategies for enhancing the fruit coloration of red-fleshed kiwifruit.

## 2. Results

### 2.1. Effects of Fruit Bagging on Anthocyanin Accumulation in ‘Hongyang’ Kiwifruit

**Figure 1:**
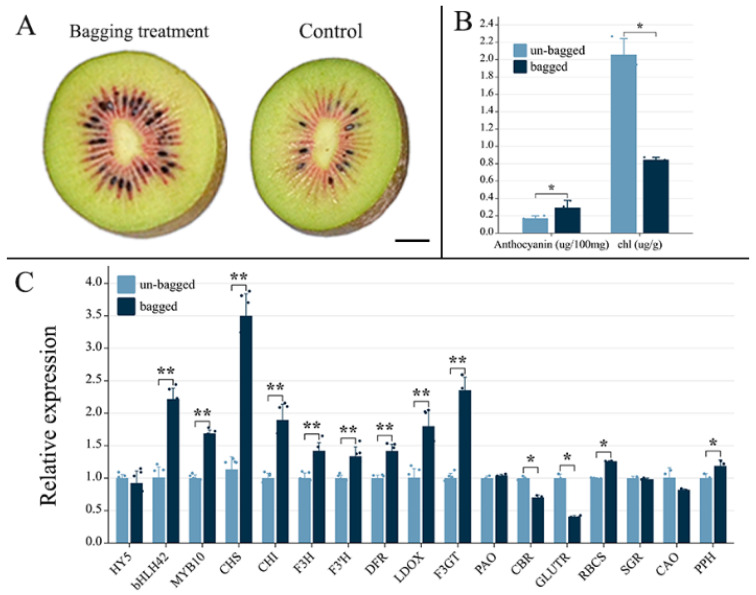
Phenotypes, anthocyanin and chlorophyll contents, and related gene expression of bagged and unbagged ‘Hongyang’ kiwifruit: (**A**) bisected fruits of bagged and unbagged kiwifruit harvested at 130 days after flower (DAF); (**B**) the contents of anthocyanin and total chlorophyll (chl) in the flesh of bagged and unbagged kiwifruit harvested at 130 DAF; and (**C**) expression level of light response gene (*HY5*), anthocyanin biosynthesis and transcriptional regulation factor genes (*CHS*, *CHI*, *F3H*, *F3′H*, *DFR*, *LDOX*, *F3GT, bHLH42*, and *MYB10*), and chlorophyll biosynthesis (*PAO*, *CBR*, *GLUTR*) and degradation-related genes (*RBCS*, *SGR*, *CAO* and *PPH*) of bagged and unbagged kiwifruit harvested at 130 DAF. Abbreviation: *HY5*, (ELONGATED HYPOCOTYL5); *CHI*, chalcone isomerase; *CHS*, chalcone synthase; *F3H*, flavonoid 3-hydroxylase; *F3′H*, flavonoid3′-hydroxylase; *DFR*, dihydroflavonol 4-reductase; *LDOX*, leucoanthocyanidin dioxygenase; *F3GT*, UDP-flavonoid 3-O-glycosyltransferase. *CAO*, chlorophyll-a oxygenase; *CBR*, chlorophyll-b reductase; *GLUTR*, glutamyl tRNA reductase; *PAO*, pheophorbide a oxygenase; *PPH*, pheophytin pheophorbide hydrolase; *RBCS*, small subunit of ribulose-1,5-bisphosphate carboxylase; *SGR*, stay-green. *, *p* ≤ 0.05; **, *p* ≤ 0.01.

After bagging treatment, the inner pericarp of ‘Hongyang’ kiwifruit exhibited significantly deeper pigmentation phenotypes than unbagged fruits (Figure 1A). Additionally, bagged fruits displayed significantly higher total anthocyanin (0.29 ± 0.08 mg/100 g FW) and lower chlorophyll (0.85 ± 0.03 mg/100 g FW) levels than unbagged fruits (0.17 ± 0.03 mg/100 g FW and 2.06 ± 0.18 mg/100 g FW). Regarding gene expression, no significant difference was observed in the expression level of the light response gene *HY5* between bagged and unbagged fruits. However, with respect to anthocyanin synthesis, bagged kiwifruits exhibited significantly higher expression levels of two key transcription factor genes (*bHLH42* and *MYB10*) as well as seven important structural genes (*CHS*, *CHI*, *F3H*, *F3′H*, *DFR*, *LDOX*, and *F3GT*), than unbagged ones (Figure 1C). Furthermore, two chlorophyll-biosynthesis-related genes, *CBR* and *GLUTR,* showed decreased expression levels in bagged fruit, whereas two chlorophyll-degradation-related genes, *RBCS* and *PPH*, demonstrated increased expression levels in bagged fruits relative to unbagged fruits; these changes may contribute to the enhanced green pigmentation observed in unbagged kiwifruits. Nevertheless, no significant differences were detected in the expression levels of *PAO*, *SGR*, or *CAO* between bagging-treated and untreated kiwifruits. Collectively, these results indicate that the application of bagging can effectively accelerate anthocyanin synthesis within the pericarp tissue of ‘Hongyang’ kiwifruit and suggest the involvement of regulation-related genes *bHLH42* and *MYB10* in transcriptional regulation processes associated with anthocyanin synthesis induced using this treatment.

### 2.2. Effect of High Postharvest Temperature on Anthocyanin Accumulation in ‘Hongyang’ Kiwifruit

**Figure 2:**
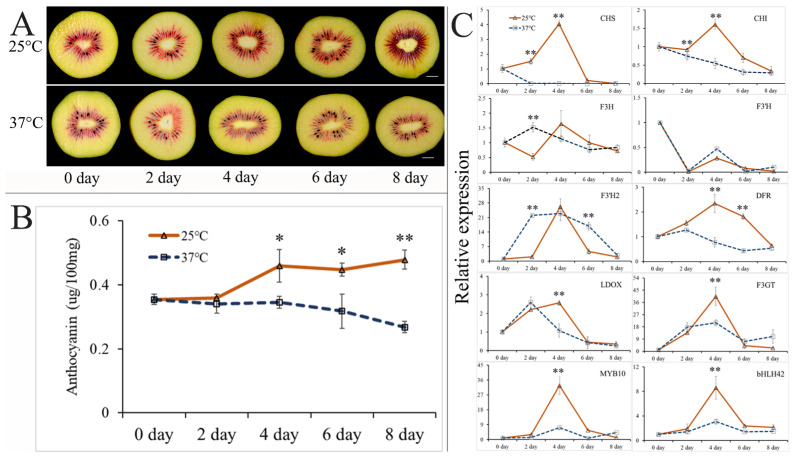
Phenotypes, anthocyanin contents, and gene expression levels of ‘Hongyang’ kiwifruit stored at 25 °C and 37 °C after harvest: (**A**) cross-cutting phenotypes of ‘Hongyang’ kiwifruit after 0, 2, 4, 6, and 8 days of storage at 25 °C and 37 °C. Scale represents 1.0 cm; (**B**) anthocyanin contents in ‘Hongyang’ kiwifruit after 0, 2, 4, 6, and 8 days of storage at 25 °C and 37 °C; and (**C**) expression levels of genes involved in anthocyanin metabolism in ‘Hongyang’ kiwifruit after 0, 2, 4, 6, and 8 days of storage at 25 °C and 37 °C. Results are presented as the mean ± SD of three replicates. *, *p* ≤ 0.05; **, *p* ≤ 0.01.

To investigate the impact of high postharvest temperatures on anthocyanin accumulation in ‘Hongyang’ kiwifruit during storage, two temperature treatments (25 °C and 37 °C) were implemented based on our previous study [26]. As depicted in Figure 2A, the red pigment in the inner pericarp of ‘Hongyang’ kiwifruit gradually darkened with increasing storage time under 25 °C treatments, whereas at 37 °C, the red pigmentation gradually decreased. The anthocyanin content in ‘Hongyang’ kiwifruit significantly increased during storage at 25 °C starting at 4 days, but slightly decreased at 37 °C (Figure 2B). Significant differences in the anthocyanin content between the treatments at 25 and 37 °C were observed on days 4, 6, and 8 of storage.

To identify the key genes involved in the downregulation of anthocyanin synthesis induced by high postharvest temperature, qRT-PCR was performed to detect the expression levels of eight key structural genes in the anthocyanin biosynthesis pathway (*AcCHS*, *AcCHI*, *AcF3H*, *AcF3′H*, *AcF3′H2*, *AcDFR*, *AcLDOX*, and *AcF3GT*) and two important transcription factor genes (*AcMYB10* and *AcbHLH42*) (Figure 2C). During the 25 °C storage period, the expression levels of all the genes initially increased, followed by a decline, reaching their peak expression after 4 days of storage, except for *AcCHI*, *AcF3H,* and *AcF3′H*; during 37 °C storage, no consistent gene expression pattern was observed, suggesting disordered anthocyanin synthesis when the postharvest temperature is high. Notably, significantly higher expression levels of *AcCHS*, *AcCHI*, *AcDFR*, *AcLDOX*, *AcF3GT*, *AcMYB10*, and *AcbHLH42* were observed after 4 days of storage at 25 °C than at 37 °C (Figure 2C). As the first significant difference in anthocyanin content between the two temperature treatments was detected on day 4 of storage (Figure 2B), these findings suggest that a critical timing for the response of kiwifruit to high postharvest temperature occurs within the initial 4 days of storage. Furthermore, only one significant difference being found in the expression levels of *AcLDOX*, *AcF3GT*, *AcMYB10*, and *AcbHLH42* on day 4 of storage between the two temperature treatments implies their role in responding to high-temperature stress and influencing postharvest anthocyanin synthesis. To further detect the interaction among these anthocyanin-related genes, Pearson’s correlation coefficient (r) test was performed using the qRT-PCR data (Figure S2). In general, the expression levels of *AcCHS*, *AcCHI*, *AcF3H*, *AcDFR*, *AcLDOX*, *AcF3GT*, *AcMYB10* and *AcbHLH42* exhibited statistically significant correlations (*p* < 0.05). Notably, the expression level of *AcMYB10* exhibited a strong positive correlation with that of *AcbHLH42* (*r* = 0.96), while their expressions also demonstrated strong positive correlations with *AcCHS* (*r* = 0.87 and 0.82, respectively) and *AcF3GT* (*r* = 0.89 and 0.84, respectively). Collectively, these results indicate that elevated postharvest temperatures have a detrimental impact on the synthesis of anthocyanins in ‘Hongyang’ kiwifruit; *AcLDOX*, *AcF3GT*, *AcMYB10*, and *AcbHLH42* may play crucial roles in regulating the temperature-induced accumulation of anthocyanins.

### 2.3. Interaction between AcMYB10 and AcbHLH42

**Figure 3:**
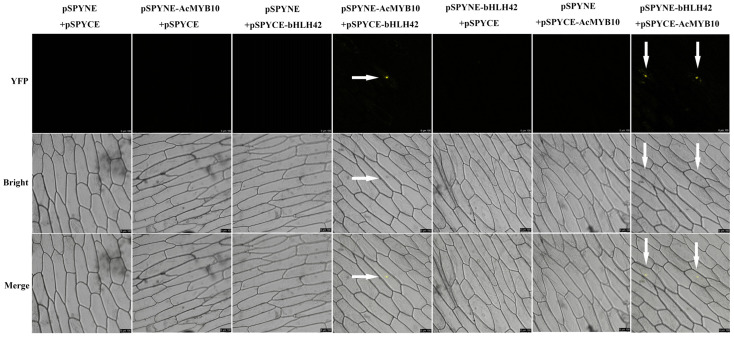
Bimolecular fluorescence complementation (BiFC) illustration of the interaction between AceMYB10 and AcbHLH42 in onion (*Allium cepa*) epidermal cells. Note: pSPYNE and pSPYCE vectors were used as the negative controls; YFP fluorescence was detected 2 days after transfection; photos were taken on a fluorescence microscope at a magnification of 200×; arrows point to the locations of the yellow fluorescence.

**Figure 4:**
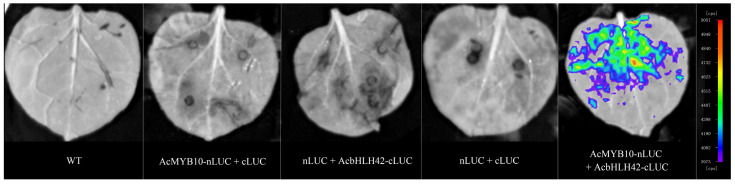
Bimolecular luminescence complementation (BiLC) illustration of the interactions between AcMYB10 and AcbHLH42 in tobacco (*N. benthamiana*) leaves. Note: The four constructs were used as negative controls: WT, AcMYB10-nLUC+cLUC, nLUC+AcbHLH42-cLUC, and nLUC+cLUC. AcMYB10-nLUC and AcbHLH42-cLUC were combined at 1:1 (*v*/*v*). The images were captured with a charge-coupled device camera at 3 days post inoculation (dpi); scale at the bottom right of the picture represents 1 cm.

Considering the significantly correlated expression of *AcbHLH42* and *AcMYB10* observed, we investigated the interactions between AcMYB10 and AcbHLH42 using bimolecular fluorescence complementation (BiFC) assays. Two fusion protein vectors, pSPYNE-AcMYB10 (or pSPYNE-AcbHLH42) and pSPYCE-AcbHLH42 (or pSPYCE- AcMYB10), were constructed and co-transformed into onion epidermal cells (Figure 3). As a result, a strong yellow fluorescent signal was observed in the nucleus transformed with both pSPYNE-AcMYB10 + pSPYCE-AcbHLH42 and pSPYNE-AcbHLH42 + pSPYCE-AcMYB10, as well as nuclear localization. No fluorescent signal was detected in the cells co-transformed with negative controls (pSPYNE-AcMYB10 + pSPYCE, pSPYNE-AcbHLH42 + pSPYCE, pSPYCE-AcbHLH42 + pSPYNE and pSPYCE-AcMYB10 + pSPYNE) or the two empty vectors (pSPYCE + pSPYNE) (Figure 4). These analyses indicate that both AcMYB10 and AcbHLH42 are nuclear proteins and are able to physically interact in plant cells.

To confirm this interaction, *AcMYB10* was fused to the N-terminal of LUC (AcMYB10-nLUC), and *AcbHLH42* was fused to the C-terminal of LUC (AcbHLH42-cLUC). Then, the constructs were transiently expressed in *Nicotiana benthamiana* leaves. The results showed that only leaves co-transformed with AcMYB10-nLUC and AcbHLH42-cLUC produced a strong LUC signal (Figure 4). No fluorescence signal was detected in the WT (blank control) or in AcMYB10-nLUC+cLUC, nLUC+AcbHLH42-cLUC or nLUC+cLUC) (Figure 4). The results further confirmed the interaction between AcMYB10 and AcbHLH42.

### 2.4. AcMYB10 and AcbHLH42 Regulate Promoter Activity of AcLDOX and AcF3GT

**Figure 5:**
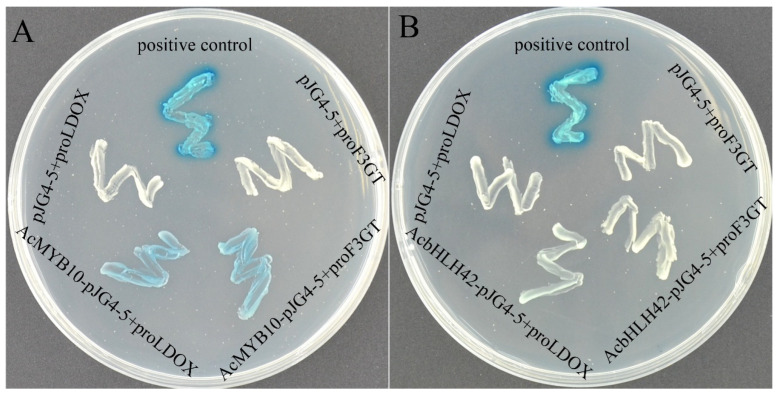
Y1H assay of AcMYB10 and AcbHLH42 on the promoters of AcLDOX and AcF3GT: (**A**) Y1H assay showing AcMYB10 binds to the promoters of *AcLDOX* (AcLDOX pro::Lacz) and *AcF3GT* (AcF3GTpro::Lacz); and (**B**) Y1H assay showing bHLH42 does not bind the promoters of *AcLDOX* (AcLDOX pro::Lacz) and *AcF3GT* (AcF3GTpro::Lacz). Note: positive control: “p53::LacZ + pJG-p53”; negative control: “bait + pJG4-5” and co-transformants (bait + prey) on SD/-Trp/-Ura medium supplemented with X-gal for 3 days.

Given the synergistic expression patterns of *AcMYB10*, *AcbHLH42*, *AcLDOX* and *AcF3GT*, Y1H assays were performed to investigate the transcriptional activation of AcMYB10 and AcbHLH42 on the promoters of *AcLDOX* and *AcF3GT* (Figure 5). The results showed that all of the yeast cells grew well on SD/-Trp/-Ura media, whereas only the positive control and bait vectors AcLDOXpro::Lacz and AcF3GTpro::Lacz co-transformed with the prey vector pJG-AcMYB10 had blue cells on media supplemented with X-gal, showing the AcMYB10-promoted expression of LacZ driven by the AcLDOX and AcF3GT promoters (Figure 5A). AcbHLH42 could not directly bind to the promoters of *AcLDOX* or *AcF3GT* (Figure 5B). From the above results, we concluded that AcMYB10 and AcbHLH42 bind to each other and AcMYB10 could directly binds to the promoter of *AcLDOX* and *AcF3GT*, thereby regulating anthocyanin biosynthesis.

## 3. Discussion

In this study, our findings demonstrated that bagging treatment significantly increased anthocyanin accumulation, reduced chlorophyll content, and promoted the expression level of anthocyanin synthesis genes in the inner pericarp of ‘Hongyang’ kiwifruit, which is in accordance with the previous studies on kiwifruit [39], apple [40] and peach [41]. However, despite being reported as a light-responsive and versatile transcription factor that directly binds to, and induces, the expression of certain MYB transcription factors, or that activates the expression of biosynthetic enzyme genes in conjunction with *PIF3* activity along with other MYB factors [24,42,43,44,45], HY5 did not demonstrate any significant synergistic effect on fruit-bagging-induced anthocyanin synthesis in ‘Hongyang’. This suggests that the observed increase in anthocyanin synthesis in bagged fruit may be independent of light. Therefore, we propose that bagging treatments might enhance anthocyanin content through comprehensively modifying the microenvironment surrounding ‘Hongyang’, including reducing ambient temperature and humidity. In addition to anthocyanin accumulation, the effect of bagging treatment on the other qualities of ‘Hongyang’ kiwifruit needs more in-depth and comprehensive research, as previously reported [16].

While the positive regulatory role of low temperature in promoting anthocyanin synthesis in horticultural crops has been widely acknowledged [46,47,48,49,50,51,52], relatively limited attention has been devoted to investigating the impact of high temperature on anthocyanin accumulation [53]. The findings from our previous study [26] demonstrated that maintaining a temperature ≤25 °C during fruit ripening on the tree ensures the functional biosynthesis of anthocyanins in ‘Hongyang’. However, the microenvironment surrounding kiwifruit during development is inherently unstable, and kiwifruit is commonly harvested prematurely and subjected to postharvest ripening treatments in most commercial contexts in China. Based on this, we investigated the impact of high postharvest temperatures on anthocyanin synthesis. The results demonstrated that, similar to preharvest conditions, high postharvest temperatures negatively affect anthocyanin synthesis. Although the results of gene expression analysis in our study suggested that high temperatures negatively regulate the genes involved in anthocyanin synthesis, further research is required to determine whether these elevated temperatures induce anthocyanin degradation. Additionally, considering the predominant green-fleshed nature of most kiwifruit cultivars, further investigations are warranted to determine whether post-harvest high-temperature conditions delay (as observed in Thai lime) [54] or enhance (as seen in banana) [55] chlorophyll degradation.

Currently, accumulating evidence strongly supports the dominant role of the MYB–bHLH–WD40 complex in regulating anthocyanin biosynthesis across the plant kingdom [34]. In kiwifruit, the interactions between AcMYB123 and AcbHLH42 [38], AcMYBF110 and AcbHLH1/4/5 [56], as well as AcMYB10 and AcbHLH42 in our present study [36] positively regulate the expressions of *AcF3GT* and *AcANS* (*AcLDOX*), thereby facilitating anthocyanin biosynthesis in the inner pericarp of ‘Hongyang’ kiwifruit. Therefore, based on the findings of this study, we hypothesize that the regulation of enhanced anthocyanin biosynthesis induced via fruit bagging or via 25 °C postharvest storage treatment (compared with 37 °C) may also involve a similar mechanism (Figure S3). However, the regulation of individual or specific anthocyanin accumulation remains poorly understood, necessitating the utilization of advanced measurement techniques (such as HPLC, LCMS, or metabolome profiling) in future investigations. Furthermore, given the abundance of MYB orthologs and paralogs in plant genomes, further investigation is warranted to elucidate their synergistic, distinct, redundant, or competitive roles in anthocyanin synthesis. Additionally, it is crucial to explore their regulatory mechanisms including the identification of interacting bHLH partners.

## 4. Materials and Methods

### 4.1. Plant Materials and Sample Treatment

The bagging experiment was conducted on 8-year-old female vines of *A. chinensis* cv. ‘Hongyang’ at the Cangxi Kiwifruit Research Institute in Sichuan Province, China (105.96° E, 31.76° N). Nine vines with similar growth vigor and yield potential were divided into three replicates, each containing three vines. At 40 days after flowering (DAF), thirty fruits from each plant were carefully enclosed in custom-made kiwifruit paper bags (Jinguonong Packaging Materials Co., Ltd., Yantai, China) with a single layer of yellow outer surface and a black inner surface (150 mm × 180 mm). The fruits were collected at 130DAF while the unbagged fruits were collected at the same time and served as controls. The flesh of each fruit (excluding the fruit axis) was diced and immediately frozen using liquid nitrogen before being securely stored at −80 °C for further analysis. For the postharvest treatment, a total of 180 fruits, harvested at 130DAF and exhibiting identical ripeness and size, without any physical damage or bacterial infection, were sampled. The fruits were randomly divided into two treatment groups: one was stored at 37 °C and the other at 25 °C, with three replicates for each temperature condition. Fruit samples were collected at 0, 2, 4, 6, and 8 days of storage for each treatment group, respectively, with six fruits per time point. The flesh (excluding the fruit axis) of each fruit was chopped and treated with liquid nitrogen before being stored at −80 °C for subsequent measurement.

### 4.2. Determination of Anthocyanin and Chlorophyll Content

The total anthocyanin content was extracted and determined following the method described by Shin et al. [45] and Lim et al. [57]. In brief, the sample was ground in liquid nitrogen, weighed accurately (0.10 g), and mixed with 600 µL of 1% hydrochloric methanol buffer. The mixture was then shaken overnight (8 h) at 4 °C in darkness using a shaker (TS-2102C, JTLIANGYOU, Changzhou, China). To each sample, 200 µL of double-distilled water and 200 µL of chloroform were added before centrifugation at 12,000 rpm for 10 min. The absorbance values were measured at wavelengths of 530 nm and 657 nm using a multifunctional full-wavelength enzyme labeler (Infinite M200 PRO, TECAN, Männedorf, Switzerland) by an absorbing supernatant with a volume of 200 µL. Finally, the anthocyanin content was calculated as A530 − 0.33 × A657.

Chlorophyll a and b were extracted and determined using the methods described by Hiscox and Israelstam [58]. In summary, the sample was triturated with liquid nitrogen in mortars. A precise weight of 0.10 g (±0.04 g) of powder was diluted to 5.0 mL dimethyl sulfoxide in a 15 mL centrifuge tube and transferred to a vibration incubator at 28 °C under dark conditions and at a speed of 200 rpm/min for 72 h. After centrifugation, the absorbance values of the supernatant extract were measured at wavelengths of 663 nm and 645 nm using a spectrophotometer. The content of Chl a, Chl b, and total Chl were calculated as follows: Chl a = (12.7 × OD663 − 2.69 × OD645) × VT/W × 1000 × VS; Chl b = (22.9 × OD645 − 4.68 × OD663) × VT/W × 1000 × VS; Chl = Chla + Chlb.

### 4.3. Determination of Gene Expression Levels

The total RNA was extracted using a plant RNA extraction kit (RC401, Vazyme, Nanjing, China) according to the manufacturer’s instructions. Briefly, 1 μg of RNA was treated with DNase I and reverse transcribed at 37 °C using a reverse transcription kit (AE341-02, TransGen Biotech, Beijing, China). qRT-PCR was performed on a Quant Studio 6 Flex system (ThermoFisher Scientific, Carlsbad, CA, USA), following the manufacturer’s instructions for AceQ qPCR SYBR Green Master Mix (Q131-02, Vazyme, Nanjing, China). Based on the previous reports [2,24,36,38], we selected seventeen genes involved in light response, anthocyanin synthesis, and chlorophyll synthesis and degradation for determination of their expression levels (Table S1). Among them, *HY5* is a key gene related to light response [24]; *bHLH42* and *MYB10* are transcription factors that regulate anthocyanin synthesis; *CHS*, *CHI*, *F3H*, *F3′H*, *DFR*, *LDOX* and *F3GT* are structure genes on the anthocyanin biosynthesis pathway [59]; and *PAO*, *CBR*, *GLUTR*, *RBCS*, *SGR*, *CAO* and *PPH* are structural genes related to chlorophyll synthesis and degradation. Achn107181 (Actin) was conducted as the endogenous control [60]. All the primers used in this study are listed in Table S1. The qRT-PCR procedure and expression level determination method (2^−∆∆ Ct^) were performed following the workflow reported by Yu et al. [36].

### 4.4. Bimolecular Fluorescence Complementation (BiFC) Assay

The BiFC assay was performed based on the procedures previously described [61]. Briefly, the coding sequences (CDS) of *AcMYB10* and *AcbHLH42* were cloned into a pSPYNE vector, resulting in pSPYNE-AcMYB10 and pSPYNE-AcbHLH42 encoding 155 amino acids at the N-terminal of yellow fluorescent protein (YFP). The sequences were then inserted into a pSPYCE carrier containing YFP C-terminal 83 amino acids, resulting in the generation of pSPYCE-AcbHLH42 and pSPYCE-AcMYB10. The recombinant or control vectors were transformed into *A. tumefaciens* strain EHA105 and then co-transformed into onion epidermal cells using an infiltration buffer. After 48 h invasion, the yellow fluorescence signal of YFP in the onion nucleus was observed using a confocal three-dimensional scanning microscope with excitation wavelengths set at YFP:510 nm and DAPI:488 nm (Leica-Microsystems TCS-SP8, Wetzlar, Germany).

### 4.5. Bimolecular Luminescence Complementation (BiLC) Assay

The BiLC assay was performed as previously described [62]. Briefly, the full-length ORF of AcbHLH42, excluding the stop codon, was cloned into the pCAMBIA-nLUC vector to create the fusion vector pCAMBIA-AcbHLH42-nLUC. Similarly, the complete ORF of *AcMYB10* was cloned into the pCAMBIA-cLUC vector to generate the fusion vector pCAMBIA-AcMYB10-cLUC. *Agrobacterium* cultures containing these constructs were co-transformed into tobacco leaves at a 1:1 ratio. After incubating in darkness for 12 h, plants were transferred to light conditions at 25 °C for 48 h. Transformed tobacco leaves were then soaked in a solution of 0.15 mg mL^−1^ D-Luciferin potassium (115144-35-9, Coolaber, Beijing, China) for 2–3 min before observing LUC activity using a Chemiluminescence Imaging System (NightSHADE LB985, Berthold, Germany).

### 4.6. Yeast One-Hybrid (Y1H) Assay

The Y1H assay was performed as previously described [61]. Briefly, the ~1.5 kb promoter sequence of *AcLODX* and the ~1.1 kb promoter sequence of *AcF3GT* (Figure S1) were amplified and inserted into pLacZi plasmids (Clontech; TaKaRa Bio Inc., Shiga, Japan) using EcoRI and kpnI restriction sites to generate AcLODX pro::LacZ and AcF3GT pro::LacZ constructs. The full-length CDS of *AcMYB10* and *AcbHLH42* were cloned into pJG4-5 vectors (Clontech; TaKaRa Bio Inc.) using EcoRI and XhoI restriction sites, creating pJG-AcMYB10 and pJG-AcbHLH42, respectively. The NcoI-digested AcLODX pro::LacZ and AcF3GT pro::LacZ vectors were co-transformed with the pJG-AcMYB10 and pJG-AcbHLH42 vectors into yeast strain EGY48 using a yeast transformation kit (SK2400-200T, Coolaber, Beijing, China). The transformants were cultivated on SD/-Trp-Ura dropout medium at 30 °C for a duration of 2 days. Subsequently, single colonies were streaked onto an SD chromogenic medium (X-gal) and incubated at 28 °C for a period of 2 days.

## 5. Conclusions

The application of a fruit bagging treatment significantly enhanced anthocyanin accumulation and reduced chlorophyll accumulation in the pericarp of ‘Hongyang’ kiwifruit during fruit development. The results of gene expression analysis suggested that *AcMYB10*, *bHLH42*, *AcCHS*, *AcCHI*, *AcF3H*, *AcF3′H*, *AcDFR*, *AcLDOX*, and *AcF3GT* are responsible for upregulating anthocyanin levels, whereas *AcCBR*, *AcGLUTR*, *AcRBCS*, and *AcPPH* may contribute to downregulating chlorophyll levels. The high-temperature postharvest treatment (37 °C) significantly suppressed anthocyanin synthesis compared with the control at 25 °C, as evidenced by noticeably decreased anthocyanin accumulation starting from day 4 of storage. This inhibitory effect could be attributed to the downregulation of key genes and TFs, including *AcCHS*, *AcCHI*, *AcF3H*, *AcDFR*, *AcLDOX*, *AcF3GT*, *AcMYB10*, and *AcbHLH42*. The nuclear expression of *AcMYB10* and *AcbHLH42*, as well as the interaction between their translated proteins, was confirmed through BiFC and BiLC analyses. The results of a yeast one-hybrid assay revealed that *AcMYB10* can activate the promoters of *AcLDOX* and *AcF3GT*, potentially elucidating their high expression synergy in fruit bagging and postharvest experiments. This study provides a further step in elucidating the mechanisms regulating anthocyanin synthesis induced by fruit bagging and temperature treatments in ‘Hongyang’ kiwifruit.

## Data Availability

The data presented in this study are included in the article and supplementary materials. Additional related data can be obtained by contacting the corresponding authors upon request.

## Supplementary Materials

The following supporting information can be downloaded at: https://www.mdpi.com/article/10.3390/genes15010097/s1, Table S1. Primer sequences used in this study; Figure S1. Promoter sequences of *AcF3GT* and *AcLDOX*; Figure S2. Correlation analysis of gene expression between anthocyanin synthesis genes and regulatory genes; Figure S3. Regulation model of anthocyanin accumulation pathway in bagging and storage of kiwifruit.
