# Supplementary material for: AcMYB10 Involved in Anthocyanin Regulation of ‘Hongyang’ Kiwifruit Induced via Fruit Bagging and High-Postharvest-Temperature Treatments
Source: Genes (Basel). 2024 Jan 14;15(1):97. doi: 10.3390/genes15010097 (PMC10815172; doi:10.3390/genes15010097)
Supplement: Supplementary file 1 [file genes-15-00097-s001.zip › genes-2801615-supplementary.pdf]

Table S1. Primer sequences used in this study

Experiment	Genes	Forward (5'-3')	Reverse (5'-3')	References
qRT-PCR	AcActin (Achn107181)	TGAGAGATTCCGTTGCCCAGAAGT	TTCCTTACTCATGCGGTCTGCGAT	Petriccione, M. et al. Reference gene selection for normalization of RT-qPCR gene expression data from Actinidia deliciosa leaves infected with *Pseudomonas syringae* pv. *actinidiae. Scientific Reports*, 2015, 5, 16961.
	AcHY5	ATGAGATCAGGAGAGTGCCG	AATCTCTTGAGCCGCTTGTG	Kim, S. et al. High ambient temperature represses anthocyanin biosynthesis through degradation of HY5. *Frontiers in Plant Science*, 2017, 8,1787
	AcCHS	ACAGCTTGACCACCTAAATGGGCTTA	CAAAGTGGAATAAAGCATGGCCAAT	Peng Y. et al. Differential regulation of the anthocyanin profile in purple kiwifruit (*Actinidia* species). *Horticulture Research*, 2019, 6(1): 3.
	AcCHI	GGAGGAGTTGACGGAATCCGTTG	AACTTTTCGACGGCTTTGGCCT	
	AcF3H	TGTACCCGCTCGCGATCCAG	ATTGCGTCTCTTTCGCAAGTTTCTTG	
	AcF3'H	GGTGGCGTATGCTCCGGAAGA	GTGGCCCGCACTCACCAGTG	
	AcDFR	GTCGGAGAAGCTGATTGGGATGGG	TGGCATTTCCATTGGGAGGGGTT	
	AcLDOX	AACAGTGGTCGGAACTAGGG	TCCTCCGAAACCAAGTCCTC	
	AcF3GT	TAGCCAAGCAGAGATCCGCTTCC	CAAGAATCCTTCTGGTAAGTACTGTTTCGA	
	AcbHLH42	AAGGGGAAGGCGGTGGATTCCG	GCACTGCAGCTCAACCAACG	
	AcCAO	ATCCGTTGGCACCTTGAA	GCGGCCCTCGTTTATTG	Liu Y. et al. Expression differences of pigment structural genes and transcription factors explain flesh coloration in three contrasting kiwifruit cultivars. *Frontiers in Plant Science*, 2017, 8:1507.
	AcRBCS	GTTACATGGGCCAAGAAAACT	TCAAACTCAAGGCAAGGGAT	
	AcCBR	ATCGGATTCAAGTTATCAGGTG	GTTCGTCCCAGCATTGTTTAT	
	AcGLUTR	CCGTTCCCACGATCAAGA	TGTCACTCCCGTCACATCTAAG	
	AcPPH	TGGAGGTGCTGTGGGATGA	CAAAAGATGTTGCTGGATTGG	
	AcPAO	TCCCGACCCCATTCCAA	TCAAAGGCGTCAGGCAAC	
	AcSGR	ACAAGCGACTGTGAGATGCC	GGTTTAGGGTTTGGTTTTGG	
CDS cloning	*AcBHLH42*	ATGGCGGCTCCCCCTA	TAGGGTATGATTTGGTGTATT	Wang L. et al. A MYB/bHLH complex regulates tissue-specific anthocyanin biosynthesis in the inner pericarp of red-centered kiwifruit *Actinidia chinensis* cv. *Hongyang*. *the Plant Journal*, 2019, 99: 359-378.
	AcMYB10	TAATAATCACTTGGTGTAGTGTAGG	GCAGACTCAACTCCATCTAATATTT	Peng Y. et al. Differential regulation of the anthocyanin profile in purple kiwifruit (*Actinidia* species). *Horticulture Research*, 2019, 6(1): 3.
Promoter cloning	*ProAcF3GT*	CTTGGACGAACCAGTGAG	AATGTACCGACAAGGAGC	Yu, M. et al. Light-and temperature-induced expression of an R2R3-MYB gene regulates anthocyanin biosynthesis in red-fleshed kiwifruit. *International Journal of Molecular Sciences*, 2019, 20, 5228.
	ProAcLDOX	GGGCATGATGGTTGGTTA	TACAGACGGAAGGCGACA	

>pAcF3GT-1058

TGGACGAACCAGTGAGTACATATATATACATGTATATGGAGGAAGAGAATAGTTTAATTTAGAAACA
AGTTAAATACAGAATATTTTAATAAAGTAGAGAAAAATAGAGAGTATTGTTGGATAACGTGTTTAAAA
AGTAATTTCTTATAAATAGGGAATGTACTTTTTTGATAAAATTTAAATAAAGAATGAGGTTTATTGTTG
AAGATGCTCTAATCATGTACTTTGGGTATGTTTGAACATTAAGTTTAGTTTCATGGAAGGGGAAAAATT
TGCTGAATTAAGGTTTGCTAGAAAAGTGGGCATGAGTCAAACTAGAAGCTTTTGGGGGGATGGGGAT
TTAGTAGCAAATGCTATGTTTGGAATCAGCTTATTAGATTAGTAAATTTTTTAAAAATTCCTTTAAGTTA
AAAAAAAAAAGGAAAAATCAATGCTGTATAGTAAAAAGGTAAGATGCTTAAACCAAAATTTGTTGGG
AATATTTGTATTTGTGCTTTTTTTTTTTTAACGAATTACTACTTTGCCTGTTAGGCAATGTTCTTCAATTTT
TTGAAAAATAATTTTTTTAAAGCGAGGCATTTTTTCATCTTAGTCATCCTTAGTCATATGGATAAATAAG
TTGTTTAACGGATGGAGCAAATCCGAAATATTGTCACAAACAAAGTAGACCTTTTCTTGTTGGCTTAC
AATCCAAAAAGCAAACAAGGCCTAAACTTTGTTAGAGCTGCACAATGCAAACACACAGTTACAAGCT
GACTTGTGTAGTCGAAGACATGCGAACCCCCAATACCTTTCCTCAAATACACAACCCCCAATCAAGT
ACACAAGCCACGGGCCAACCACCGTTCTCTCTCTCTCATACCTTATAAAACAGCTTTTCTTACATTC
ATTTCGATATATCACTTTTCTTAGTACTAGCCTCCTGAGCTTTGTGGACTTTTATAGGTCCTTTAACTCT
TTTTCAGTCAACCAAAGCCAAGCCAAGCCTAGCCTAGCCACCAAAATTCGCCTGAAAACACCACAAG
CTAGACCCAAAATCAAAGCCTTAAAAAAACCATG

> pAcLDOX-1500

TGTAGATCCAGTTTTTAGGTAAATTTCACCAATGGTCCCTGTAGTTATACCAAAGTTTCACTTTGGTCC
TTATATTTTCAATTTAAAACAATGACAACCCTATAATTTTAATTTAGTTATAACGTAGTCTCTCCGTCCA
TGCCTTTTAAAATTTTGGATGGAAAGTGAGCATGCAAGCTCATGTAAACCTCTTTAAAGGGTAATTAT
ACAACCATTAGTCCCAGTAGTTTGGCCAAAGTTTCACTTTGGTCCTTATAAATGCCACTTGAGTTTTTA
CATGTTTTTTGTTCATCGAAATTGTTGATGGGTATTGACAGATGAATTATATTAAAACTAAGTTAAAAC
TTCAGAATCCTAATTGTTCAAACTAAAATTATAGTTACTTAAGTGAAATTTTTGTCAGATTAGAGGGAC
TAATGGTGAAACTTACCCTTGAAAATTCAAATTAGGATGGGATTGAACATGAGGAAAGAAAGAGAAG
TGAATGGTAGACATGACTACATGAGTAATTGAAAGTCTTAATTATTCCAACAGTCCTAACATTTCTTTG
CTAATTTAAGCTAATGTACCACAATTTTCCTCATTCTTTGAAATCGAACAAATTCAAGTCGTTTTCTTTT
TTCTTATACCTTTTTTTCCCCACAAAACCGAACATAAAAATTTCAACGGTAACAGTAAGACTCAGATTC
AAAAGTCGTGCTCAAGAAGAGAGTGCACGGCTCAACTTAGAGACTATTGATATTGAACATTTGGTATT
TGGTCTTTTACGTAAAAATGTGGTTGATAGATATCGATTGAGTCGTGATTGTTGTGAGGTACGTGAAC
AAATATTAAGTGTAACGATGATACTATTTTACGTGGTATTGGTATCACTAGCCAATAAAAATTAATGAA
ATTTCCGGCTAACCCAATGATATGTTATTGTGAATACCGCAAAATAAAATTTATCATTATTCAGTTACT
ACCAATATACCCAACTAGCTAAAATTGGGGAATATGCAATGAGTGGGCAAGCAATGGAAATAGTAA
AAAAAAATTAATAATTTTGAGTTGATCTATGAAAAAAAACAGAAGTGGAAATGTATAACGGTTGAGAT
CATGTAAAAAATTACTATTTTACCCTTACAATTAATTTAGAAAGTTTTTACGAACCATTATAGTTGATTG
ACAATAATGATAATTTAATTAAGAACAAGAAAAAGTGTAAAGGGTGACATTGGATCTCTATCTCACGA
CGGTTAGACCACCTCGTGCTTGTGCCATCCAGTCAAGTCCACTCCCCACGTGAGAAACTGATTCAAA
ACCACCTCTCTCACTTTTAAGTTGTTAGCTAGCCCTAACCCCTCACCAGTCAGCACCTTCTTGAAAAA
ACCAACACATTGCATAGTTGAAGCAACAAAAACCTCCTATAAAACTCCCCAAACCCTAATCACAACT
CACATAATTGTCACAAATTCACACATAAGTACCGAATTACATTATAATTTAGGTGTCGAAAAAAATG

Figure S1. The promoter sequences of *AcF3GT* and *AcLDOX*
Note: cis-acting elements are indicated in green.

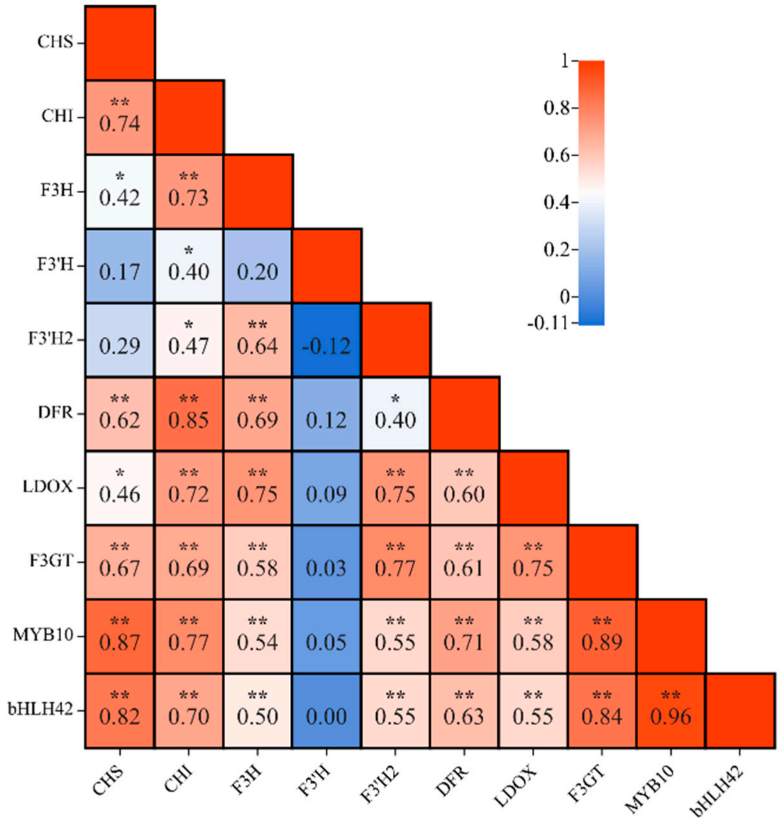

Figure S2. Correlation analysis of gene expression between anthocyanin synthesis genes and regulatory genes. Note: * and ** represent statistical significance at $p \leq 0.05$ and $p \leq 0.01$ levels, respectively.

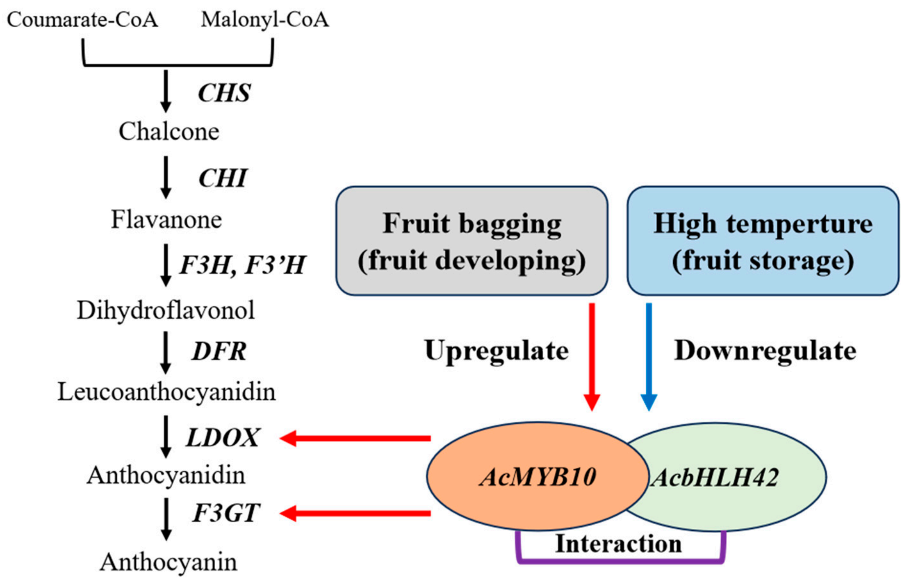

Figure S3. Regulation model of anthocyanin accumulation pathway in bagging and storage of kiwifruit.
